# The Anaphylactic and Anti-allergenic Properties of Shuanghuanglian: A Review

**DOI:** 10.2174/0113862073328626241107044327

**Published:** 2025-01-13

**Authors:** Xin Jiang, Ji Li, Xiaohui Yao, Hao Ding

**Affiliations:** 1 Baoying People's Hospital, Yangzhou, 225800, China

**Keywords:** Shuanghuanglian, allergy, asthma, rhinitis, dermatitis, therapeutic effects

## Abstract

Shuanghuanglian (SHL) and its primary constituents have demonstrated protective effects against allergenic diseases. This review examines the anaphylactic and anti-allergenic activities of SHL and its constituents. We also discuss potential avenues for future research, particularly regarding the expansion of the clinical applications of SHL formulations (oral or nebulized) for the treatment of allergenic disorders.

For this review, we searched the PubMed, Web of Science, and China National Knowledge Infrastructure databases for relevant publications. Additionally, details of the essential active components and target genes of SHL were obtained from the Traditional Chinese Medicine Systems Pharmacology database (TCMSP), and information on allergy-related genes was collected from the GeneCards and Online Mendelian Inheritance in Man (OMIM) databases. Lists of both the SHL target and disease-related genes were imported into the ‘Draw Venn Diagram’ tool on the website (http://bioinformatics.psb.ugen/webtools/Venn/). A protein–protein interaction network for SHL and disease targets was constructed with reference to the Search Tool for the Retrieval of Interacting Genes/Proteins (STRING) database, and the potential pathways were identified based on Kyoto Encyclopaedia of Genes and Genome enrichment analyses.

The allergenic reactions induced by SHL injection (intravenous) and its main constituents (intraperitoneal or intravenous injection) have been verified in animal experiments. Furthermore, the protective effects of SHL injection (intraperitoneal) and its individual Chinese herb components (intragastric administration), namely, *Flos Lonicerae*, *Radix Scutellariae*, and *Fructus Forsythiae*, as well as their main constituents (intraperitoneal or intragastric administration), have been verified in asthma, rhinitis, atopic dermatitis, and both IgE- and non-IgE-mediated systemic allergic responses. The network pharmacology analysis revealed that the therapeutic effects of SHL might be primarily mediated through the regulation of the IL-17 and TNF-α signalling pathways and Th17 cell differentiation.

Accumulated research data provide a theoretical basis for the clinical application of SHL (*via* extravascular routes) in the treatment of allergenic diseases.

## 1 INTRODUCTION

Shuanghuanglian (SHL), a well-established traditional Chinese medicinal preparation listed in the *Chinese Pharmacopoeia* (2015 Edition), is derived from Yinqiaosan in the renowned Qing dynasty masterpiece ‘Item Differentiation of Warm Febrile Diseases’ [1]. The SHL preparation comprises extracts obtained from *Flos Lonicerae* (*Jinyinhua* in Chinese), *Radix Scutellariae* (*Huangqin*), and *Fructus Forsythiae* (*Lianqiao*). Currently, more than 120 components have been identified in SHL injection [2, 3], with baicalin, chlorogenic acid, and *Forsythia suspensa* being notable marker compounds. Since the 1970s, SHL has been approved as an antimicrobial treatment for patients with acute respiratory infections, effectively addressing symptoms such as sore throat, cough, and fever [4, 5]. It has been viewed as a broad-spectrum antiviral agent that can target respiratory syncytial virus [6], influenza virus [7-9], coxsackie A/B virus [10, 11], and human adenovirus III [12]. Notably, combining orally administered SHL with standard care has been found to improve clinical outcomes in patients with COVID-19 [13]. SHL has also been shown to inhibit the key 3C-like protease of SARS-CoV-2 *in vitro* [14]. With respect to bacterial infections, SHL has been demonstrated to have anti-inflammatory and antioxidative properties in lipopolysaccharide-induced murine acute lung injury [15], and it also inhibits the production of inflammatory mediators (TNF-α, IL-6, and IL-1β) by suppressing the p38- and ERK1/2-mediated AP-1 pathway in lipopolysaccharide-stimulated murine alveolar macrophages [16]. Furthermore, its immunomodulatory activity has proven beneficial in the treatment of respiratory infections. The administration of SHL can enhance natural killer (NK) cell activity, promote the production of alpha-interferon, and increase the rate of lymphocyte transformation [6]. It has also been used to treat viral myocarditis [17], viral encephalitis [18], and aconitine-induced arrhythmia [19].

Clinically, SHL can be administered *via* multiple routes in a range of different formulations, including oral liquids, soft capsules, injections, nebulized inhalation, and rectal suppositories. Injectable preparations of SHL, which offer superior rapid action and high bioavailability, are important formulations in clinically critical fields. Given its excellent efficacy, injectable SHL has been recommended as a national hospital emergency medicine by the State Administration of Traditional Chinese Medicine since 1992 [20]. However, adverse drug reactions (ADRs) have been reported in approximately 3.25% of patients receiving SHL injections [21], with 70% of these ADRs being classified as hypersensitivity reactions [22]. For example, Tang *et al.* have reported that among 11,001 patients who received SHL injections, 182 experienced skin and mucosal allergies, such as urticaria and rashes, and in 14 cases, this treatment resulted in anaphylaxis [23]. The IgE- and non-IgE-mediated allergenic reactions induced by the injection of SHL and its main constituents have also been verified both *in vitro* and *in vivo* [24-26]; these reactions have contributed to serious complications [27]. The findings of recent animal studies have provided evidence to indicate that SHL and its components can have protective effects against asthma, rhinitis, atopic dermatitis, and IgE- and non-IgE-mediated allergenic reactions [28-30]. Nevertheless, despite these important findings, the mechanisms underlying SHL-induced hypersensitive reactions and the potential anti-allergenic pathways remain unclear. In this review, we examine the anaphylactic and anti-allergenic properties of SHL and its components, focusing in particular on conditions such as asthma, rhinitis, and atopic dermatitis. Furthermore, with the aim of providing a theoretical basis for enhancing the clinical application of SHL in treating allergic diseases, we use a network pharmacology approach to analyse the potential anti-allergenic pharmacological mechanisms of SHL.

## 2 THE MAJOR CHEMICAL CONSTITUENTS AND QUALITY CONTROL OF SHL

In the Chinese Pharmacopoeia, baicalin, chlorogenic acid, and phillyrin are designated as marker compounds for the quality control of SHL. Additionally, liquid chromatography fingerprinting and similarity analyses have quantified and identified seven key components, namely, chlorogenic acid, caffeic acid, galuteolin, baicalin, luteolin, baicalein, and wogonin, for the quality evaluation of SHL injections [31, 32]. Similarly, Si *et al.* developed an approach that combines linear principal component analysis with quantitative fingerprinting to determine five components (phillyrin, baicalin, chlorogenic acid, neochlorogenic acid, and cryptochlorogenic acid) for the quality evaluation of SHL solutions and suspensions [33]. We also examined the concentrations of 10 components in injectable SHL and acquired similar fingerprints based on high-performance liquid chromatography (HPLC) analysis. The major components of SHL include baicalin, forsythiaside B, forsythiaside A, rutin, and neochlorogenic acid (1,078.23, 47.85, 40.97, 13.93, and 10.6 μg in 1 mL of SHL injection, respectively) [13]. In Fig. (**1**), we show the chemical structures of the main compounds comprising SHL. A potential SHL compound structure file was gathered and downloaded from the SciFinder Database.

## 3 SHL-INDUCED HYPERSENSITIVITY REACTIONS

The hypersensitive responses induced by SHL are primarily linked to its injectable formulations (solution and powder). In 2001 and 2009, the National Adverse Drug Reaction Monitoring Centre issued public warnings regarding the potential for severe ADRs when administering SHL injections, including anaphylactic shock, dyspnea, and exfoliative dermatitis [4]. In 2018, the China Food and Drug Administration issued a notice contraindicating its use in pregnant women and children under 4 years of age [21]. The incidence of ADRs with SHL injection is approximately 3% [4], with hypersensitivity reactions accounting for more than 70% of these ADRs [27]. Moreover, the risk of such ADRs increases when SHL injection is used in combination with other drugs, especially penicillin [34]. However, although reactions such as local skin and mucosal issues, including rashes and itching, are commonly reported, the incidence of systemic anaphylaxis is comparatively low [4]. In contrast, no serious adverse events have been reported in patients treated with oral SHL preparations [13].

Acute allergenic reactions, including type I allergies and pseudo-allergenic reactions to a variety of traditional Chinese medicinal injections, have been reported previously [35, 36]. In IgE-mediated type I allergies, some drugs have the ability to bind covalently to proteins, forming hapten-carrier complexes, and thus elicit the crosslinking of high-affinity IgE receptor-bound IgE with multivalent antigens, initiating the activation of mast cells and basophils [37, 38]. Pseudo-allergenic reactions result from the direct triggering of mast cell and basophil degranulation in a non-IgE-dependent manner [39]. Reports of SHL-induced ADRs have revealed that most SHL injection-mediated hypersensitivity reactions in patients occur in response to an initial exposure, with a majority of these responses occurring within 1 h following drug administration [40]. On the basis of these clinical observations, most authors subscribe to the view that SHL can cause pseudo-allergenic reactions [22, 24, 41].

### 3.1 SHL-Induced Pseudo-Allergenic Reactions

The mechanisms underlying SHL-induced pseudo-allergenic reactions involve the release of histamine and activation of the complement pathway. SHL injection with a single dose of 150 to 600 mg/kg has been shown to cause vascular leakage, oedema, and exudates in the ears, lungs, and intestines of mice, whereas in human umbilical vein vessel endothelial cells, stimulation with SHL injection for 1 h has been observed to induce a rearrangement of the actin cytoskeleton and promote the formation and distribution of stress fibres [24]. Furthermore, intravenous bolus injection of SHL in a dose range of 100 to 400 mg/kg has been found to result in a reduction in mean arterial pressure and an increase in plasma histamine levels in rats. The finding that the hypotension induced by SHL is not completely blocked by a histamine H_1_ receptor antagonist tends to indicate that the hypotension caused by SHL may be partially mediated by histamine [41]. In a further study, Gao *et al.* reported that SHL injection directly activates the blood complement component C5, subsequently stimulating mast cells or basophils to release histamine [22]. Consistent with these findings, SHL injections have been shown to promote increases in the levels of complement components C4d and Bb in dogs [27].

Baicalin has been identified as a primary constituent responsible for the pseudo-allergenic reactions induced by SHL [20]. For example, baicalin has been shown to dose-dependently induce rat basophilic leukaemia cell (RBL-2H3) degranulation and β-hexosaminidase release [25], promote histamine release and intracellular calcium influx in LAD2 human mast cells, and contribute to increases in paw thickness and Evans blue exudation in mice following baicalin injection [42]. However, it has been established that baicalin does not induce local swelling in Mpgprb2-knockout mice, thereby indicating that baicalin induces Mrgprb2-dependent pseudo-allergy in mice [43]. On the basis of intraplantar injection and application of the Evans blue exudation method, Gao *et al.* examined eight constituents from an extract of *Lonicerae Japonicae Flos*, and *Fructus Forsythiae*, namely, cryptochlorogenic acid, forsythigenol, isochlorogenic acids B and C, eugenol, quercitrin, d-(-)-quinic acid, and luteolin. They accordingly showed that these constituents could induce significant vasopermeability in response to the initial injection in mice, whereas the effects of these eight constituents could be blocked using the C5a antagonist PMX53 [22]. Chlorogenic acid and its isomers have been demonstrated to markedly promote the proliferation of popliteal lymph node cells [44]. Chlorogenic acid has also been reported to alter the membrane fluidity and degranulation of RBL-2H3 rat basophilic leukaemia cells by increasing calcium ion influx and F-actin depolymerisation [45]. Wang *et al.* examined the pseudo-allergenic reaction of three phenolic acids (chlorogenic acid, neochlorogenic acid, and cryptochlorogenic acid) in honeysuckle. Wang *et al.* found that chlorogenic and cryptochlorogenic acids both promoted increases in the plasma serotonin and β-hexosaminidase levels in guinea pigs and elicited the degranulation of RBL-2H3 cells, whereas they detected no similar reactions in response to treatment with neochlorogenic acid [46]. However, Wu *et al.* demonstrated that it was macromolecules in lower purity chlorogenic acid extracts (purity of ≤40%), rather than the chlorogenic acid per se, that were closely associated with anaphylaxis, as no similar pseudo-allergenic reactions were observed in response to treatment with high-purity chlorogenic acid samples (purity of ≥92%) [47]. Of the main constituents of *Fructus Forsythiae* extracts, forsythoside A and forsythoside B have been established to contribute to SHL injection-induced pseudo-allergenic reactions *via* activation of the RhoA/ROCK signalling pathway [48]. In addition, it is speculated that macromolecular substances (>10 kDa) and Tween 80 could be the main harmful factors causing SHL-induced anaphylactic or pseudo-allergenic reactions [49-51].

### 3.2 SHL-Induced IgE-Mediated Anaphylaxis

Wang *et al.* identified haptens in SHL by combining biochemical detection with HPLC-mass spectrometry and found that 10 compounds (baicalein, 3,5-dicaffeoylquinic acid, 3-caffeoylquinic acid, 1,5-dicaffeoylquinic acid, baicalin, 3,4-dicaffeoylquinic acid, 4,5-dicaffeoylquinic acid, forsythiaside A, 1,3-dicaffeoylquinic acid, and scutellarin) showed higher absorbance values than those detected prior to sensitization, providing evidence for their potential sensitization effects [26]. In further studies, Gao *et al.* injected (i.p.) a combination of baicalin and bovine serum albumin into guinea pigs on days 1, 3, and 5 of treatment, inducing anaphylactic shock following a challenge on day 33. Both specific IgE and IgG were detected in the sensitized serum, and peritoneal mast cell degranulation rates were higher than those in the control group. Moreover, ilea isolated from the sensitized guinea pigs contracted significantly after being challenged with baicalin and human serum albumin [52]. In Table **1**, we summarize the mechanisms associated with SHL-induced hypersensitivity reactions.

## 4 SHL EXERTS ANTI-ALLERGENIC EFFECTS

Most of the clinical reports on SHL have been published in Chinese, among which several clinical studies focusing on its treatment for bronchial asthma were conducted in the 1990s. In this regard, SHL injection and aerosol inhalation have been demonstrated to ameliorate the acute symptoms during asthma attacks. One notable study involved 30 patients who received an intravenous drip of injectable SHL (60 mg/kg) mixed with a 5% glucose solution without antibiotics. The outcomes assessed after 7 days revealed that 20 patients were cured and nine improved, whereas the remaining patients showed no response [53]. SHL injection has been used to treat different skin diseases in 50 patients, achieving a cure in 27 patients with allergenic skin diseases, including urticaria, drug dermatitis, lichen urticatus, and eczema [54]. However, the quality of these studies was often limited, as they relied on subjective measures such as cure rates, which can contribute to ambiguity and bias.

Recently, some authors conducting *in vivo* and *in vitro* experiments relating to allergenic asthma and anaphylaxis have reported that SHL has a protective effect against allergenic diseases. Specifically, SHL injection was found to reduce ST-induced T helper (Th) 2 immunity by inhibiting the basophilic Ca^2+^-NFAT pathway, thus suppressing early IL-4 release prior to specific immunoglobulin E (sIgE) synthesis and inhibiting basophile activation in the presence of sIgE [28]. SHL injections have also been demonstrated to inhibit the degranulation of RBL-2H3 cells and reduce phorbol 12-myristate 13-acetate (PMA)/A23187-induced inflammatory cytokine production (TNF-α, IL-6, and prostaglandin E2) in human basophilic KU812 cells. Moreover, *in vivo* results revealed that SHL injection contributed to reducing paw swelling in mice during both the immediate (1 h) and late (24 h) phases following the allergen challenge [55]. Simultaneously, by activating the mitochondrial calcium uniporter, SHL reduces Ca^2+^ levels, thereby stabilizing mast cells and thus exerting marked anti-allergic activity [56]. Furthermore, using an allergen-induced asthma model, Gao *et al.* demonstrated that SHL injections can alleviate eosinophilic airway inflammation and airway hyperresponsiveness [29]. Indeed, the extracts of single Chinese herbs, including *Flos Lonicerae*, *Radix Scutellariae*, and *Fructus Forsythiae*, and their main constituents have been verified in asthma, rhinitis, atopic dermatitis, and both IgE- and non-IgE-mediated systemic allergenic responses.

### 4.1 Anti-Allergenic Effects of Scutellaria Baicalensis and its Constituents

The plant *Scutellaria baicalensis* is a fundamental constituent of a number of traditional Chinese medicinal preparations that are used to treat respiratory infections [57]. TNF-α and IL-1, produced by mast cells and tissue macrophages, have been identified as the main pro-inflammatory cytokines associated with innate immune responses [58, 59]. In response to treatment with an ethanol extract of *S. baicalensis* (28.0 mg/100 g body weight), a 6.6% inhibition of the passive cutaneous anaphylaxis reaction was observed based on the amounts of dye observed in anti-DNP IgE-injected rats. In addition, treatment with a similar extract has been found to suppress IL-8 and TNF-α production and MAP kinase expression in human mast cells (HMCs) stimulated with PMA plus A23187, as well as compound 48/80-induced mast cell degranulation and histamine release from rat peritoneal mast cells [30, 60].

The anti-allergenic effects of baicalin, the primary constituent of *S. baicalensis*, have been reported in several *in vivo* and *in vitro* studies. For example, Ma *et al.* found that treatment with baicalin (10, 25, and 50 mg/kg) resulted in a significant increase in pulmonary dynamic compliance and a reduction in pulmonary resistance and eosinophil counts in an asthma model established *via* an intraperitoneal injection of ovalbumin (OVA) in 60- female BALB/c mice [61]. Furthermore, treatment with baicalin has also been shown to reduce the serum and BALF levels of OVA-specific IgE, IL-4, IL-17A, and C-C motif chemokine (CCL) 19 production in bronchoalveolar lavage fluid [61, 62], and to regulate the imbalance of Th17/Treg responses in mice with allergenic asthma [63]. In airway remodelling based on chronic inflammation, baicalin (25, 50, and 100 mg/kg) treatment was found to suppress the expression of transforming growth factor-β1, IL-13, and vascular endothelial growth factor and inhibited the activation of the extracellular signal-regulated kinase pathway in 70 female BALB/c mice exposed to OVA [64]. Park *et al.* reported the molecular targets of baicalin, demonstrating reduced TNF-α levels and monocyte influx into BALF in the context of allergenic asthma, which could be partially attributed to its ability to inhibit phosphodiesterase 4 [65]. Baicalin has been shown to inhibit nasal symptoms *via* the inhibition of autophagy and regulation of the balance of Th17/Treg cytokines by reducing the levels of IL-17A and retinoic acid-associated nuclear orphan receptor (ROR)-γt, and promoting increases in the levels of IL-10 and Foxp3 in 42 male BALB/c mice with OVA-induced allergic rhinitis [66]. Baicalin has also been established to alleviate intestinal pathological changes in rats with food allergy [67] and 2,4-dinitrofluorobenzene-induced contact hypersensitivity in mice [68]. Similarly, baicalin, baicalein, and wogonin have been found to have protective effects against allergenic asthma, atopic dermatitis, and food allergies [30, 69, 70]. In Fig. (**2**), we summarize the anti-allergenic effects of *S. baicalensis* and its constituents.

### 4.2 Anti-Allergenic Effects of Flos Lonicerae and its Constituents

An extract of *Flos Lonicerae* has been shown to inhibit passive cutaneous anaphylaxis induced by OVA in mice [71]. Similar to prednisolone, a water-soluble polysaccharide from the flower buds of *Flos Lonicerae* has been found to induce significant inhibition of allergenic contact dermatitis-associated mouse ear swelling by 32.4%, 42.9%, and 49.6% at doses of 20, 40, and 80 mg/kg, respectively. This polysaccharide was also found to reduce serum levels of IgE and histamine and tissue TNF-α levels in 40 female ICR mice with picryl chloride-induced allergenic contact dermatitis [72].

Chlorogenic acid has been established as the major phenolic compound in *Flos Lonicerae* extracts, and the release of the atopic dermatitis-related chemokine C-X-C motif chemokine ligand (CXCL) 8 is suppressed in IL-31- and IL-33-activated eosinophils as well as in eosinophil-dermal fibroblasts co-cultured with chlorogenic acid [73]. The effects of chlorogenic acid on allergic rhinitis-related parameters in OVA-induced mice have also been previously reported. For example, the treatment of 60 male BALB/c mice with chlorogenic acid (50, 100, and 200 mg/kg) regulated Th1/Th2-related cytokines by inhibiting the levels of IL-4, IL-5, and IL-13 while increasing the production of IFN-γ and IL-12 in the nasal lavage fluid and nasal tissues, thereby ameliorating nasal mucosal inflammation and mucosal thickness [74]. Chlorogenic acid has also been found to regulate Th17 responses by reducing the ratio of CD4^+^ IL-17^+^ Th17 cells relative to CD4^+^ T cells in peripheral blood and the mRNA and protein levels of IL-17A and ROR-γt *in vivo* [75].

A further constituent identified in *Flos Lonicerae* is luteolin, a member of the flavone group of compounds, which has regulatory effects on mast cell-mediated inflammatory diseases and allergies. Studies show it inhibits IgE-mediated mast cell activation [76] and reduces the release of β-hexosaminidase and histamine from HMCs (LAD2) stimulated with either substance P (SP) or IgE/anti-IgE [77]. Luteolin has also been found to suppress the release of histamine from compound 48/80-stimulated rat peritoneal mast cells [78]. Luteolin has the capacity to attenuate allergenic nasal and lung inflammation, with mononuclear cells derived from peripheral blood of allergic rhinitis patients treated with luteolin showing a reduced percentage of CD4^+^ IL-4-secreting cells [79]. Furthermore, some authors have reported that luteolin attenuates airway inflammation in OVA-induced asthmatic mice. For example, Kim *et al.* investigated the mechanisms associated with anti-asthmatic effects subsequent to treatment with luteolin (20 mg/kg), and found that luteolin induced a larger number of CD4^+^ CD25^+^ Treg cells in anti-CD3/anti-CD28-stimulated murine splenic CD4^+^ T cells. The transfer of CD4^+^ CD25^+^ Treg cells into 40 asthmatic male BALB/c mice was observed to reduce eosinophilia, whereas this suppression of eosinophilia was found to be abolished in CD25-depleted OVA-sensitized mice [80]. Furthermore, Wang *et al.* reported the regulatory mechanisms of luteolin underlying autophagy in allergenic asthma, which was proposed to involve activation of the PI3K/Akt/mTOR signalling pathway and inhibition of the Beclin-1-PI3KC3 complex [81]. Luteolin has also been found to reduce the expression of IL-33, IL-1β, IL-6, and IL-8 in canine atopic dermatitis [82], whereas caffeic acid [83, 84] and rutin [85, 86] have been reported to have potential preventive and protective effects against allergenic asthma and dermatitis. In Fig. (**3**), we summarize the anti-allergenic effects of *Flos Lonicerae* and its constituents.

### 4.3 Anti-Allergenic Effects of Fructus Forsythiae and its Constituents

Oral administration of an 80% methanol extract of *Forsythia suspensa* (100 mg/kg) alleviated passive cutaneous anaphylaxis (PCA) activity and reduced the levels and numbers of plasma histamine and mast cells, respectively, in the small intestines of weaned piglets with soybean allergy [87]. Sung *et al.* used a 70% ethanol extract of *Forsythia suspensa* (400 μg/mouse) to treat atopic dermatitis-like skin lesions in 28 NC/Nga mice and found that similar to tacrolimus, the extract attenuated the atopic dermatitis symptoms, including an elevated dermatitis severity score, ear thickness, and infiltration of inflammatory cells in the skin lesions, while ameliorating oedema and erosion. Additionally, it reduced IL-4 and IFN-γ mRNA levels in the thymus and activation of regulated chemokines (TNF-α, IL-4) and regulated upon activation, normal T-cell expressed and secreted (RANTES) in skin tissues [88].

Forsythiaside and phillyrin are the main polyphenolic compounds isolated from *Fructus Forsythiae*, and Qi *et al.* found that a forsythiaside-rich extract from *Forsythiae Fructus* can inhibit Mas-related G-protein coupled receptor- and IgE/FcεRI-mediated degranulation in LAD2 and RBL-2H3 cells. Moreover, they established that the underlying mechanisms involved a reduction in cytosolic Ca^2+^ levels mediated by an enhanced mitochondrial uptake of Ca^2+^ [89]. Among forsythiasides, forsythiaside A (15, 30, and 60 mg/kg) has been shown to inhibit OVA-induced AHR, reduce inflammatory cell counts, and suppress IL-4, IL-5, and IL-13 production in bronchoalveolar lavage fluids, as well as reduce NF-κB activation in 60 BALB/c mice [90]. *Forsythia suspensa* fruit extracts and their constituent matairesinol have been established to have anti-allergenic effects in an allergenic dermatitis mouse model [91], whereas phillyrin, a novel cyclic AMP phosphodiesterase 4 inhibitor, might serve as a useful candidate agent for treating allergenic dermatitis [92]. In Fig. (**4**), we summarize the anti-allergenic effects of *Fructus Forsythiae* and its constituents, and in Table **2**, we list the anti-allergenic effects and mechanisms of SHL and its constituents.

**Fig. (5):**
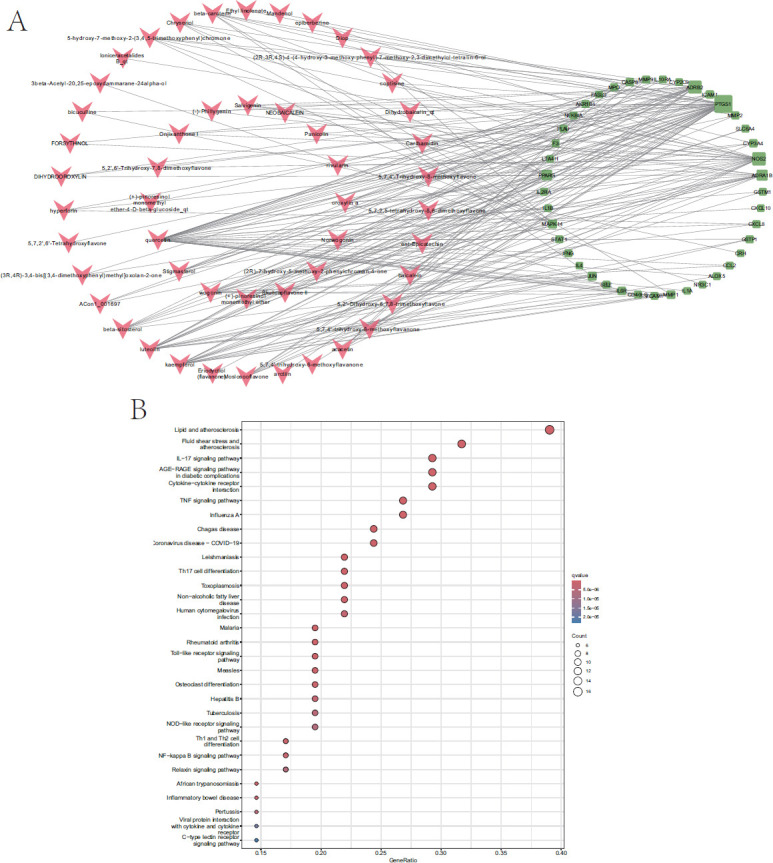
Network pharmacology analysis of Shuanghuanglian. (**A**) Compound-target network. (**B**) KEGG enrichment analysis. The Y-axis represents significant KEGG pathways and the X-axis represents the ratio of enriched targets in a pathway to all common targets. The size of the nodes shows count of targets, and gradient of color represents the adjusted *P* value.

Furthermore, to analyse the disease–compound–target network associated with SHL, we adopted a network pharmacology approach, constructing an SHL target-allergenic disease-related gene set consisting of 41 target genes and 50 components of SHL (Fig. **5A**). Based on protein–protein interaction (PPI) analysis, we identified the top 10 target genes as *IL-1β*, *CXCL8*, *CCL2*, *IL-4*, *ICAM-1*, *IFNG*, *MMP-9*, *JUN*, *PPARG*, and *IL-1α*. Detailed information regarding the degree values of the 41 genes within the PPI network can be found in Supplementary Information Table **S1**. The top 30 KEGG signalling pathways were obtained and constructed based on *P-*values. It is well established that IL-17 and TNF-α are key cytokines involved in the pathophysiology of allergic diseases. IL-17, primarily produced by Th17 cells, activates multiple signalling pathways, including NF-κB and MAPK, promoting the expression of chemokines and adhesion molecules that facilitate neutrophil and eosinophil recruitment to inflamed tissues. This, in turn, contributes to inducing airway hyperreactivity and mucus overproduction, which are considered hallmark features of asthma and allergic rhinitis [93, 94]. Similarly, TNF-α, produced by various immune cell types, orchestrates inflammatory responses by activating NF-κB signalling, leading to the expression of pro-inflammatory cytokines and enhancing Th2 responses. Moreover, synergistic interactions between IL-17 and TNF-α contribute to amplifying the allergic response, as these cytokines can promote the differentiation of naïve T cells into Th2 and Th17 cells, which exacerbate airway inflammation [95]. Furthermore, clinical analyses indicate that by inhibiting autophagy to modulate the differentiation of Th17 and T regulatory (Treg) cells, the administration of baicalin could represent an effective treatment for allergic rhinitis [66]. Additionally, chlorogenic acid has been shown to suppress the release of pro-inflammatory cytokines, such as IL-6, as well as CCL7 and CXCL8 chemokines, in eosinophil-dermal fibroblast co-cultures treated with IL-31 and IL-33 [73]. Furthermore, luteolin has been found to inhibit the secretion of inflammatory cytokines, including IL-1β and TNF-α, from human mast cells (HMC-1) stimulated with phorbol myristate acetate combined with calcium ionophore A23187 [78]. Moreover, peripheral blood mononuclear cells derived from allergic rhinitis patients treated with luteolin showed a reduced percentage of CD4^+^ IL-4-secreting cells and a reduced production of Th2 cytokines [79]. Based on these findings, it is suggested that SHL exerts its therapeutic effects primarily *via* the modulation of IL-17 and TNF-α signalling pathways, along with Th17 cell differentiation (Fig. **5B**).

A total of 82 compounds derived from the three constituent herbs comprising SHL were obtained from the Traditional Chinese Medicine Systems Pharmacology database ((TCMSP, http://www.tcmspw.com/tcmsp.php), and we employed ADME evaluation systems to identify the effective compounds and their targets, with a drug-likeness of ≥0.18 combined with an oral bioavailability of ≥30% [96]. Initially, after removing the duplicated genes, we retrieved 230 target genes from the target information database in TCMSP. Allergy-related genes were obtained from the GeneCards (score >10) (https://www.genecards.org/) and OMIM (https://www.omim.org/) databases using the keywords ‘allergenic asthma’, ‘atopic dermatitis’, ‘allergic rhinitis’, and ‘food allergy’, with 359 directly related genes after manually removing of duplicates. Both the SHL target and disease gene lists were imported into the ‘Draw Venn Diagram’ tool (http://bioinformatics.psb.ugen/webtools/Venn/). The results were subsequently imported into the STRING database to facilitate the construction of the interactive PPI network of SHL and disease targets. As a minimum interaction threshold, we used the “Medium confidence of more than 0.4” setting, and Cytoscape 3.8.0 was used to produce visual network diagrams. Moreover, we selected the topmost 10 targets by computing the degree values using the CytoHubba plugin in Cytoscape 3.8.0 [97].

(A) A disease–compound–target network. The green rectangles represent genes, and the red arrows represent active constituents. (B) Kyoto Encyclopedia of Genes Genome (KEGG) pathway analysis was performed using the R package ‘clusterProfiler’ to predict potential pathways. A *P*-value of less than 0.05 was considered to be indicative of a significant difference.

## CONCLUSION AND FUTURE PERSPECTIVES

Although Shuanghuanglian is used extensively for its therapeutic benefits, its administration can have certain undesirable effects. Documented cases of allergic reactions include skin rashes, itching, and more severe hypersensitivity reactions [4, 23]. Notably, it is the intravenous injection of SHL preparations that is the primary cause of SHL-induced anaphylaxis, while administration *via* other routes rarely causes serious adverse reactions. The main factors contributing to SHL-induced anaphylaxis are as follows. (1) The main constituents of injectable SHL, such as baicalin, cryptochlorogenic acid, and forsythosides, can promote pseudo-allergenic reactions or IgE-mediated anaphylaxis. (2) Owing to difficulties in obtaining pure preparations, along with their poor stability and solubility, there is an increased likelihood that injected SHL will cause allergic responses [36]. (3) A high incidence of serious adverse reactions has also been observed following the combined administration of injectable SHL and antibiotics, which could be attributable to drug-drug interactions or the presence of insoluble particles. (4) Individual factors, including genetic, pathological, and other biological differences, might contribute to increased susceptibility. In contrast, compared to intravenous administration, delivery *via* alternative extravascular routes is generally associated with fewer adverse reactions, as these types of delivery may reduce the risk of anaphylaxis. Moreover, given the relative ease of administration, using these alternative routes tends to improve patient compliance. Consequently, unless intravenous injection is necessary, it is generally preferable to administer SHL *via* oral or other extravascular routes. Recent studies revealed that nebulized SHL has the advantages such as rapid onset, prolonged lung retention, and a better safety profile compared to intravenously delivered SHL. Thus, nebulized SHL could serve as a viable alternative to injection [98]. Nanoparticles have been studied for their application in drug delivery [99], as they offer benefits such as high bioactivity, enhanced penetration, and low toxicity. Based on these considerations, future studies should focus on improving the purification, stability, and solubility of SHL and developing new drug formulations to determine the most effective and safe delivery methods.

Currently, the primary frontline clinical medications for treating allergic diseases include antihistamines, corticosteroids, leukotriene receptor antagonists (LTRAs), immunotherapeutics, and biologics. However, the efficacy and safety of these medications can vary, and long-term use could lead to resistance and serious adverse reactions, including osteoporosis, mental disorders, allergic reactions, and neurotoxicity. Consequently, there is an urgent need to develop highly effective and low-toxicity traditional medicines. Accumulated research findings have verified that SHL and its main constituents show considerable potential as anti-allergenic agents. In this study, we propose a theoretical rationale for the clinical application of SHL in the treatment of allergenic diseases. Compared with conventional treatments, SHL offers several potential advantages for the treatment of allergenic diseases, including lower toxicity and a reduced risk of serious long-term side effects. SHL could also represent a complementary or alternative option, particularly for patients who are intolerant to conventional therapies. However, it is important to take into consideration some of the limitations of the SHL research conducted to date. Notably, much of the clinical research on SHL in the treatment of allergenic diseases is of generally low quality. Moreover, translating the findings based on animal studies to human applications poses several challenges. Key obstacles include the physiological differences between species, which can often manifest as differences in drug metabolism and efficacy and disparities in immune responses [100]. Additionally, the complexity of human diseases often surpasses that which can be replicated in animal models, thereby potentially limiting the relevance of findings obtained in animal studies [101]. Moreover, to address these challenges, we suggest the adoption of the following strategies. First, it would be desirable to develop humanized animal models that better mimic human biology, thereby improving translational relevance. Second, integrative research approaches should be implemented, combining *in vitro* and *in silico* models to complement the *in vivo* animal data. Furthermore, employing early-phase clinical trials can provide key data on human responses, thereby contributing to the refinement and guidance of subsequent research. Research should also focus on examining the clinical application of SHL in the treatment of human asthma, rhinitis, or dermatitis, including, for example, a comparative study of the use of orally administered SHL solutions in the treatment of high T2-type (Th2-mediated) asthma and low T2-type (Th17-mediated) asthma, or the therapeutic effects of SHL (*via* the percutaneous route) on dermatitis.

There is also a need for better basic research regarding the anti-allergenic effects of the clinical application of SHL and, in particular, mechanistic studies of these effects of SHL. In this regard, receptor-ligand docking, a structure-based computer-assisted approach that has applications in the design of drugs, can be used to predict ligand binding in the function-regulating pockets of receptors. Based on the network pharmacology analysis described herein, we identified the top ten target genes of SHL constituents. Future studies should focus on assessing molecular docking structures for compounds with higher affinity for their potential targets, with subsequent validation both *in vitro* and *in vivo*. Combining computer-assisted methods will enhance the efficacy of efforts to elucidate the functions and underlying mechanisms of SHL.

## Figures and Tables

**Fig. (1):**
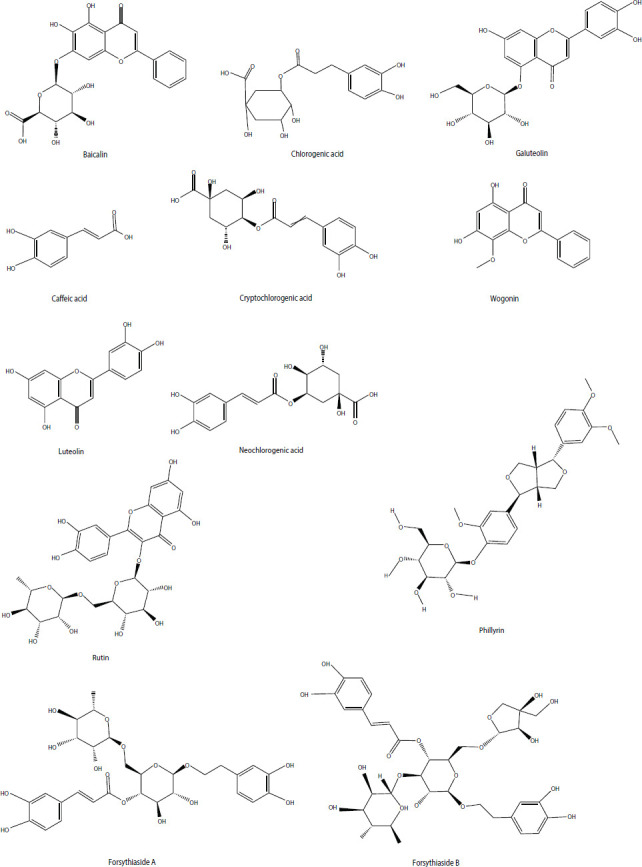
Chemical structures of the main compounds in Shuanghuanglian.

**Fig. (2):**
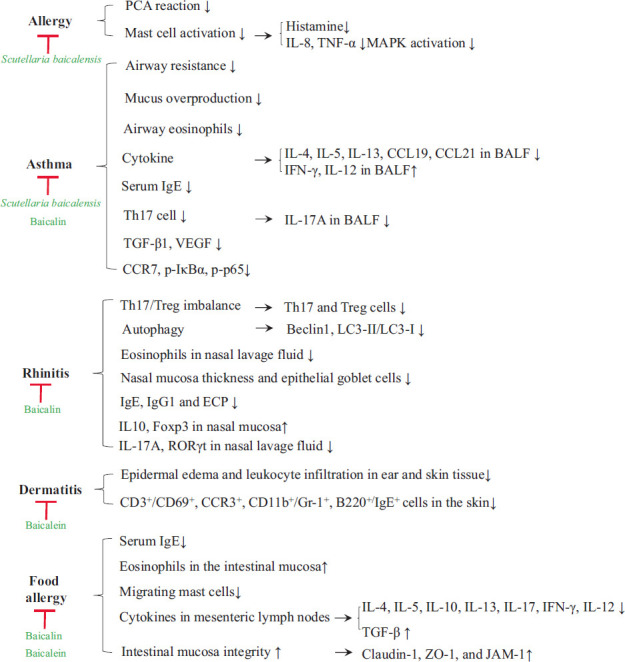
Anti-allergenic effects of *Scutellaria baicalensis* and its constituents.

**Fig. (3):**
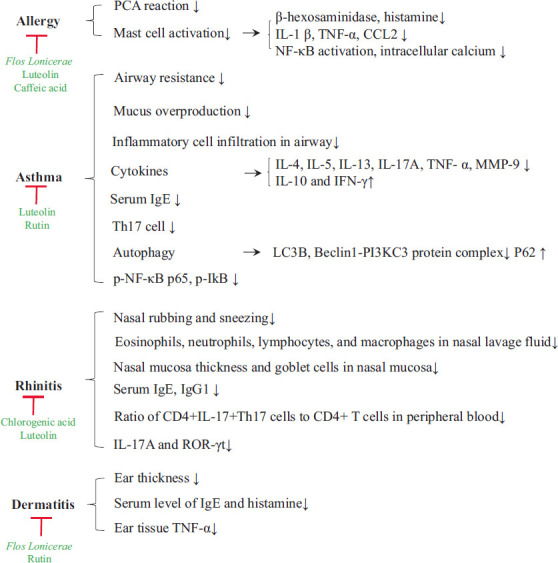
Anti-allergenic effects of *Flos Lonicerae* and its constituents.

**Fig. (4):**
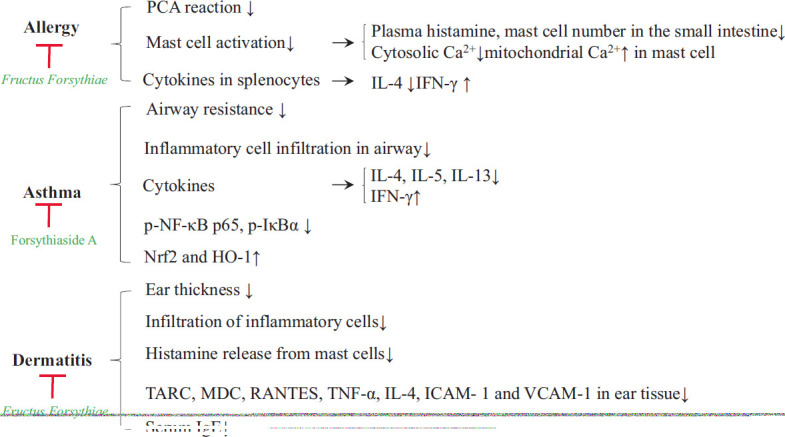
Anti-allergenic effects of *Fructus Forsythiae* and its constituents.

**Table 1:** SHL-induced hypersensitivity reactions.

**Drug**	**Dosage**	**Source**	**Effect/Result**	**References**
SHL injection	150–600 mg/kg, i.v.	Mouse	Vascular leakage↑ Oedema↑ Exudates↑ Histamine release↑ Actin cytoskeleton reorganization↑	[24]
SHL injection	100–400 mg/kg, i.v.	Rat	Mean arterial pressure↓ Plasma histamine levels↑	[41]
SHL injection	500 μL/mouse, i.v.	Mouse	Activated blood complement component C5↑	[22]
SHL injection	300 mL/h, i.v.	Beagle dog	Release of histamine↑ Mean arterial blood pressure↓ SC5b9↑ C4d and Bb↑	[27]
Baicalin	10–50 μg/mL	RBL-2H3 cells	Cell degranulation↑ β-hexosaminidase release↑	[25]
Baicalin	0.65–2.60 mg/mL, i.v.	Mouse LAD2 cells	Paw thickness and Evans blue exudation in mice↑ Histamine release and calcium influx in human mast cells (LAD2)↑	[42]
Baicalin	26.3–262.1 mg/mL, i.v.	Mouse	Does not induce local swelling in Mpgprb2-knockout mice	[43]
Cryptochlorogenic acid Isochlorogenic acid B and C	5 mg/mL, 15 μL, intraplantar injection	Mouse	Evans blue exudation in mice↑	[22]
Chlorogenic acid	20 mg/kg, i.v.	Mouse	Popliteal lymph nodes proliferation↑	[44]
Chlorogenic acid	0.2–5 mmol/L	RBL-2H3 cells	β‐hexosaminidase secretion rates↑ Cell degranulation↑ Calcium ion influx↑ F-actin depolymerization↑	[45]
Chlorogenic acid Cryptochlorogenic acid	0.16 mg/kg, i.v. 1–6.25 mmol/L	Guinea pig RBL-2H3 cells	Plasma serotonin and β-hexosaminidase levels↑ Cell degranulation↑	[46]
Forsythoside A Forsythoside B	50–100 mg/kg, i.v. 50–150 μg/mL	Mouse HUVECs	Evans blue exudation in mice↑ RhoA/ROCK↑	[48]
Baicalin	0.2% baicalin-BSA, 0.5 mL, i.p. 0.2% baicalin-BSA	Guinea pig Peritoneal mast cells	Specific IgE and IgG↑ Cell degranulation↑	[52]

**Table 2:** Anti-allergenic effects of SHL.

**Drug**	**Dosage**	**Source**	**Model**	**Effect/Result**	**References**
SHL injection	3–6 mg/kg, i.g. 0.5–2%	Mouse RBL-2H3 cells	Shrimp tropomyosin-sensitized mice Anti-shrimp tropomyosin IgE-sensitized RBL-2H3 cells	Serum sIgE, sIgG_2a_, sIgG_2b_↓ IL-4, IL-5, IL-10, IL-13 in splenocytes↓ Basophil CD200R surface expression↓ Ca^2+^-NFAT↓ IL-4↓	[28]
SHL injection	2.5–5 mL/kg, i.p. 0.5–2%	Mouse KU812 cell RBL-2H3 cells	Mouse with anti-shrimp tropomyosin serum-induced paw swelling Anti-shrimp tropomyosin serum-sensitized RBL-2H3 cells PMA/A23187-induced KU812 cells	Paw swelling ↓ Cell degranulation↓ TNF-α, IL-6, PGE_2_ in KU812 cells↓	[55]
SHL injection	150–600 mg/kg, i.g.	Mouse	Mice with shrimp protein-induced asthma	Airway hyperresponsiveness↓ Eosinophilic airway inflammation↓ IL-4, IL-5, IL-13 in BALF↓	[29]
Ethanol extract of *Scutellaria baicalensis*	100–400 mg/kg, i.g.	Mouse	Mice with OVA-induced asthma	IL-1β, IL-4, IL-5, TNF-α in BALF↓ IFN-γ, IL-12 in BALF↑ Compound 48/80-induced systemic anaphylaxis↓ Serum levels of total IgE and OVA-specific IgE, IgG1↓	[30]
Ethanol extract of *Scutellaria baicalensis*	280 mg/kg, i.g. 1, 10, and 100 μg/mL	Rat Rat peritoneal mast cells HMC-1 cells	Anti-DNP IgE-sensitized rat Compound 48/80-induced rat peritoneal mast cells PMA plus A23187-stimulated HMC-1 cells	PCA reaction in rats↓ Histamine content in rat peritoneal mast cells↓ IL-8, TNF-α, MAPK activation in HMC-1 cells↓	[60]
Baicalin	10–40 mg/kg, i.g.	Mouse	Mice with OVA-induced asthma	Airway resistance↓ Airway eosinophils↓ IL4, IL17A in BALF↓ IFN-γ in BALF↑ OVA-induced eosinophilia in lung tissues and airway tissues↓ OVA-specific IgE in BALF and serum↓ Number of Th17 cells↓	[61]
Baicalin	10–50 mg/kg, i.g.	Mouse	Mice with OVA-induced asthma	AHR, lung inflammation in mice↓ White blood cells, eosinophils, OVA-specific IgE, CCL19, CCL21 in BALF↓ Serum levels of IL-6 and TNF-α↓ Expression of CCR7 mRNA, CCR7, p-IκBα, p-p65 protein in lung tissues↓	[62]
Baicalin	10–65 mg/kg, i.g.	Mouse	Mice with OVA plus LPS-induced asthma	Serum IgE level↓ IL-17A, IL-6 in BALF↓ IL-10 in BALF↑ Peribronchial and perivascular inflammation↓ Mucus overproduction and goblet cell hyperplasia in the airways↓ STAT3, FOXP3 in lung tissues↑	[63]
Baicalin	25–100 mg/kg, i.g.	Mouse	Mice with OVA-induced asthma	IL-13 in BALF and serum↓ Expression of TGF-β1, VEGF in lung tissues↓	[64]
Baicalin	40 mg/kg, i.g. 20 μM	Mouse RAW 264.7 cells	Mice with OVA-induced asthma LPS-induced RAW 264.7 cells	Inflammatory cells, monocytes, TNF-α in BALF↓ TNF-α in macrophages↓ activity of PDE4B and PDE4A1A in macrophages↓	[65]
Baicalin	50–200 mg/kg, i.g. 100 μg/mL	Mouse Th17 and Treg cells derived from allergic rhinitis patients Peripheral blood mononuclear cells	Mice with OVA-induced allergic rhinitis Peripheral blood mononuclear cells	Th17/Treg imbalance, autophagy Th17 and Treg cells derived from allergic rhinitis patients↓ Beclin1, LC3-II/LC3-I in mice↓ P62 in mice↑ Eosinophils and epithelial cells in nasal lavage fluid ↓ Nasal mucosa thickness and epithelial goblet cells in mice↑ Serum levels of anti-OVA-specific IgE, IgG1 and ECP in mice↓ IL10, Foxp3 in nasal mucosa↑ IL-17A, RORγt in nasal lavage fluid↓	[66]
Baicalin	20 mg/kg, i.g.	Rat	Rats with OVA-induced food allergy	Serum levels of OVA-sIgE↓ Intestinal mucosa integrity↑, density of eosinophils in the intestinal mucosa↓ Mast cell integrated rate↑	[67]
Baicalin	1–5% topical creams, skin application	Mouse	Mice with 2,4-dinitrofluorobenzene -induced contact hypersensitivity	Increase in ear thickness and ear weight, thymus index, and spleen index↓ Orthokeratosis of granular layers, epidermal thickness of mouse tail skin↑	[68]
Baicalein	Hydrogel (1% baicalein), skin application	Mouse	Mice with *Dermatophagoides pteronyssinus*-induced atopic dermatitis	Epidermal oedema and leukocyte infiltration in ear and skin tissues↓ CD3^+^/CD69^+^, CCR3^+^, CD11b^+^/Gr-1^+^, B220^+/^IgE^+^ cells in the skin↓	[69]
Baicalein	20 mg/kg, i.g. 1–40μM 50–200 μM	Mouse CD4^+^ T cells human intestinal epithelial Caco-2 cells	Mice with OVA-induced food allergy	Change in rectal temperature↓ Migrating mast cells, inflammation scores in the jejunum↓ L-4, IL-5, IL-10, IL-13, IL-17, IFN-γ, IL-12 in mesenteric lymph nodes↓ TGF-β in mesenteric lymph nodes↑ claudin-1, ZO-1, and JAM-1 in intestinal epithelium↑ The population of CD4^+^Foxp3^+^ T cells differentiated from Tregs↑ Transepithelial electrical resistance value of Caco-2 cells↑	[70]
*Lonicera* extract	25–100% extract, i.g.	Mouse	Mice with OVA-induced allergy	Intestinal mast cell degranulation↓ Heat-unstable PCA activity↓	[71]
Polysaccharides from the flower buds of *Lonicera japonica*	20–80 mg/kg, i.g.	Mouse	Mice with picryl chloride-induced allergenic contact dermatitis	Ear thickness in mice↓ Serum levels of IgE and histamine↓ Ear tissue TNF-α↓	[72]
Chlorogenic acid	5–40 μg/mL	Human eosinophil	IL-31- and IL-33-treated eosinophils–dermal fibroblasts co-culture	CXCL8 release from eosinophils↓	[73]
Chlorogenic acid	50–200 mg/kg, i.g.	Mouse	Mice with OVA-induced allergic rhinitis	Nasal rubbing and sneezing↓ number of total cells, eosinophils, neutrophils, lymphocytes, macrophages, and epithelial cells in nasal lavage fluid↓ Nasal mucosa thickness↓ Serum levels of anti-OVA IgE and anti-OVA IgG1↓ Serum levels of anti-OVA IgG2a↑	[74]
Chlorogenic acid	50–200 mg/kg, i.g.	Mouse	Mice with OVA-induced allergic rhinitis	Frequencies of rubbing and sneezing of allergic rhinitis mice↓ Histopathological abnormalities and goblet cell numbers in nasal mucosa↓ Serum levels of OVA-IgE, ROR-γt, and IL-17A↓ Serum levels of IFN-γ↑ Ratio of CD4^+^IL-17^+^Th17 cells to CD4^+^ T cells in peripheral blood and the mRNA and protein levels of IL-17A and ROR-γt↓	[75]
Luteolin	1–100 μM	human umbilical cord blood-derived mast cells (hCBMCs) Human LAD2 cell	Substance P or IgE/anti-IgE stimulated LAD2 cells and hCBMCs	NF-κB activation, intracellular calcium↓ Beta-hexosaminidase, TNF and histamine secretion in LAD2 cells↓ CCL2 release from hCBMCs↓	[76, 77]
Luteolin	1–20 μM 1–20 mg/kg, i.g.	Human mast cells (HMC-1) rat peritoneal mast cells mouse	PMA plus A23187-stimulated HMC-1 Compound 48/80-stimulated rat peritoneal mast cells Pruritogen-stimulated mice	Secretion of IL-1 β, TNF-α from HMC-1↓ Histamine release from rat peritoneal mast cells↓ Scratching behaviour and vascular permeability in mice↓	[78]
Luteolin	10–30 mg/kg, i.p. 0.5–10 μg/mL	Mouse CD4 cells isolated from mouse spleens PBMCs from AR patients	Mice with HDM-induced allergic rhinitis IL-4-stimulated CD4 cells HDM-stimulated PBMCs	Allergenic symptoms in mice↓ HDM-specific IgE↓ CD4^+^ IL-4-, CD4^+^ IL-17-secreting T cells in splenocytes from AR mice↓ Infiltration of eosinophils↓ Nasal mucus production in mice↓ percentage of CD4^+^ IL-4-secreting cells in PBMCs from AR patients↓	[79]
Luteolin	20 mg/kg, i.p. 10 μg/ mL	Mouse CD4^+^CD25^-^ regulatory T cells isolated from mouse splenocytes	Mice with OVA-induced allergenic asthma CD4+CD25+ Treg cells	OVA-specific IgE in BALF↓ Infiltration of eosinophil-rich leukocyte, CD19^+^ B cells, CD4^+^ T cells, CD3-CCR3^+^, and CD11b^+^Gr-1^+^ cells in lungs↓ IL-13, TNF-α mRNA levels in lungs↓ Foxp3, IL-10, and TGF-β1 mRNA levels in lungs↑ IL- 4, IL-5, and IL-13 in BALF↓	[80]
Luteolin	10–20 mg/kg, i.g.	Mouse	Mice with OVA-induced asthma	Airway resistance in mice↓ Inflammatory cell infiltration in airway↓ Inflammatory cell count, IL4, IL5, IL13 in BALF Serum levels of OVA-sIgE↓ Expression of LC3B in lung tissues↓ Expression of P62 in lung tissues↑ Levels of p-Akt in mice↑ Beclin1-PI3KC3 protein complex↓	[81]
Luteolin	1–128 μM	Canine keratinocyte cell line (CPEK)	LPS-stimulated CPEK cells	Cell viability↓ Expression of IL-33, IL 1β, IL-6, and IL-8↓	[82]
Caffeic acid	20 mg/kg caffeic acid-assisted cross linked with OVA, i.g.	Mouse	OVA-sensitised mice	Levels of specific IgE, IgG, IgG1, and IgG2a in the mouse sera↓ serum histamine and mMCP-1 concentrations↓ IL-4, IL-5, IL-13 and IFN-γ production in stimulated splenocytes↓	[83]
Caffeic acid	100–500 mg/kg, i.g.	Mouse	Compound 48/80-sensitised mice	Scratching behaviour↓ Skin histamine content↓ Vascular permeability↓	[84]
Rutin	37.5–75 mg/kg, i.g.	Mouse	Mice with OVA plus cigarette smoke-induced asthma	Airway hyperresponsiveness↓ Lung compliance in mice↑ Eosinophil counts, lymphocytes, macrophages, and neutrophils in BALF↓ Serum and BALF levels of OVA-specific IgE↓ IL-4, IL-5, IL-13, IL-17A in BALF↓ IL-10 and IFN-γ↑ Treg cells counts↑ Th17 cells↓ Normal lung histology p-NF-kB p65, p- IkBa and TNF-a↓ MMP-9 expression in the lung tissues↓	[85]
Rutin	1–5 μg/ear, painting 5–20 μg/ear, painting	Mouse	Mice with *Dermatophagoides farinae* extract /2,4-dinitrochlorobenzene-induced atopic dermatitis Mice with 2,4-dinitrochlorobenzene-induced allergenic contact dermatitis	Ear thickness in atopic dermatitis mice↓ Epidermal and dermal thickness, serum IgE and histamine, Infiltration of mast cells into ears in AD mice↓ mRNA levels of IL-4, IL-5, IL-13, IL-31, IL-32, and INF-γ in the ear tissue in AD mice↓ Expression levels of mRNA for IL-4, IL-5, IL-10, IL-17, INF-γ and TNF-a in the ears of allergenic contact dermatitis mice↓	[86]
*Forsythia suspensa* extract	100 mg/kg, i.g.	Piglet	Piglets with β-conglycinin-induced anaphylaxis	Anaphylactic reaction↓ PCA reactions induced by unheated or heated sera from β-conglycinin-sensitized animals↓ Plasma histamine levels and mast cell numbers in the small intestines↓ IL-4 secretion from splenocytes↓ IFN-γ in splenocytes↑	[87]
*Forsythia suspensa* extract	400 μg/mouse, topical application 25–400 μg/mL	Mouse human keratinocytes	Mice with HDM-extract-induced AD TNF-α- and IFN-γ-stimulated HaCaT keratinocytes	Dermatitis severity score↓ ear thickness↓ Infiltration of inflammatory cells↓ Expression of TARC, MDC, RANTES, TNF-α, IL-4, ICAM-1 and VCAM-1 in ear tissue↓ Serum levels of IgE↓ Production of TARC, MDC, and RANTES in HaCaT cells↓	[88]
Forsythiasides-rich extract	200–600 μg/mL 50–100 mg/kg, i.p.	Rat basophilic leukaemia cell line and RBL-2H3 cells mouse	C48/80-induced mast cells and SP-challenged RBL-2H3 shrimp protein-sensitised mice	Cytosolic Ca^2+^↓ Mitochondrial Ca^2+^↑ Mast cell degranulation in mice↓	[89]
Forsythiaside A	15–60 mg/kg, i.g.	Mouse	Mice with OVA-induced asthma	Inflammatory cell infiltration and epithelial thickening in lung tissues↓ IL-4, IL-5, IL-13, and inflammatory cells in BALF↓ IFN-γ in BALF↑ Penh value↓ Phosphorylation of NF-κB and IκBα↓ Expression of Nrf2 and HO-1↑	[90]
*Forsythia suspensa* extract	25–400 μg/mL 500 μg/mouse, topical	MC/9 mast cells Mouse	Compound 48/80-induced mast cells Mice with *Dermatophagoides farinae* crude extract -induced AD	Histamine release from mast cells↓ ear thickness, dermatitis severity scores↓ Skin dryness followed by erythema, haemorrhage, oedema, scarring, Erosion, and excoriation in mice↓	[91]

## LIST OF ABBREVIATIONS

OMIM: Online Mendelian Inheritance in Man
STRING: Search Tool for the Retrieval of Interacting Genes/Proteins
TCMSP: Traditional Chinese Medicine Systems Pharmacology database
