# Supplementary material for: The Anaphylactic and Anti-allergenic Properties of Shuanghuanglian: A Review
Source: Comb Chem High Throughput Screen. 2025 Jan 13;29(3):367–86. doi: 10.2174/0113862073328626241107044327 (PMC13223432; doi:10.2174/0113862073328626241107044327)
Supplement: Supplementary file 1 [file CCHTS-29-3-367_SD1.pdf]

Supplementary Material

The Anaphylactic and Anti-allergenic Properties of Shuanghuanglian: A Review

Xin Jiang[1,#], Ji Li[1#], Xiaohui Yao[1#] and Hao Ding[1,*]

[1]*Baoying People's Hospital, Yangzhou 225800, China*

Table S1. Degree value of the 41 genes in the PPI network.

Number	Gene	Degree
1	IL1B	33
2	CXCL8	31
3	CCL2	30
4	IL4	28
5	ICAM1	27
6	IFNG	27
7	MMP9	26
8	JUN	26
9	PPARG	25
10	IL1A	25
11	MPO	24
12	VCAM1	24
13	NFKBIA	24
14	CXCL10	23
15	CD40LG	22
16	STAT1	22
17	MMP2	21
18	NOS2	19
19	SELE	19
20	FASLG	17
21	CASP8	16
22	MAPK14	16
23	MMP1	16
24	F3	15
25	NR3C1	14
26	IL2RA	14
27	ALOX5	13

28	IL6R	13
29	IL10RA	11
30	PTGS1	10
31	PLAU	10
32	ADRB2	7
33	GSTP1	7
34	CYP2C9	7
35	SLC6A4	6
36	CYP3A4	5
37	GSTM1	4
38	AKR1B1	3
39	LTA4H	3
40	CRH	3
41	ADRA1B	2
